# Supplementary material for: Remodeling articular immune homeostasis with an efferocytosis-informed nanoimitator mitigates rheumatoid arthritis in mice
Source: Nat Commun. 2023 Feb 13;14:817. doi: 10.1038/s41467-023-36468-2 (PMC9925448; doi:10.1038/s41467-023-36468-2)
Supplement: Supplementary file 1 — Supplementary Information [file 41467_2023_36468_MOESM1_ESM.pdf]

Supplementary Information for

Remodeling Articular Immune Homeostasis with an Efferocytosis-informed Nanoimitator Mitigates Rheumatoid Arthritis

Shengchang Zhang[1#], Ying Liu[1#], Weiqiang Jing[2#], Qihao Chai[3,4], Chunwei Tang[1], Ziyang Li[2,3], Zhentao Man[3,4], Chen Chen[1], Jing Zhang[1], Peng Sun[5], Rui Zhang[1], Zhenmei Yang[1], Maosen Han[1], Yan Wang[1], Xia Wei[6], Jun Li[6], Wei Li[3,4], Mohnad Abdalla[1], Gongchang Yu[7], Bin Shi[7], Yuankai Zhang[8*], Kun Zhao[1], Xinyi Jiang[1*]

[*]Corresponding author: Xinyi Jiang, Ph.D.; Yuankai Zhang, Ph.D.

Phone/Fax： +86-15662758621

Email: xinyijiang@sdu.edu.cn; drzhangyk@163.com

Contents

Supplementary Figure 1. IRF5 expression profiles in synovium.

Supplementary Figure 2. Synthesis of NBC.

Supplementary Figure 3. Synthesis of PtdSer-NBC.

Supplementary Figure 4. Zeta potential of the DOPA-stabilized nanocore, PtdSer-coroneted nanocore and siIRF5@EINI.

Supplementary Figure 5. Encapsulation efficiency of siRNA inside siIRF5@EINI at various siRNA inputs.

Supplementary Figure 6. Flow cytometry analysis of PtdSer-presenting.

Supplementary Figure 7. DLS data of the nanoimitator after triggered degradation under acidic conditions.

Supplementary Figure 8. The release of LMWH from the nanoimitator in PBS with or without 0.1 mM H_2O_2.

Supplementary Figure 9. The expression levels of CD11b and F4/80 in BMDMs analyzed by flow cytometry.

Supplementary Figure 10. Characterization FLSs.

Supplementary Figure 11. Cell-specific uptake of the nanoimitator by human peripheral blood macrophages in a coculture pattern with human FLSs.

Supplementary Figure 12. Flow cytometric analysis of the purity of neutrophils.

Supplementary Figure 13. Effective intervention by nanoimitator on inflammatory neutrophils.

Supplementary Figure 14. Quantitative analysis of neutrophils adhered to HUVECs monolayers.

Supplementary Figure 15. IRF5 expression in macrophages treated with different formulations, as detected by Western blotting.

Supplementary Figure 16. Metformin reprograms mitochondrial metabolism in macrophages and acts as mitochondrial complex I (CI) inhibitor that directly inhibits CI-derived ROS.

Supplementary Figure 17. In vivo biodistribution of nanoimitator in non-arthritic DBA/1J mice.

Supplementary Figure 18. Inflammation-targeted drug delivery of the nanoimitator.
Supplementary Figure 19. Systemic toxicity evaluation of siIRF5@EINI.
Supplementary Figure 20. Assessment of the impact of nanoimitator on the immune responses of mice to the *Candida albicans* infection.
Supplementary Figure 21. Ankle diameter change.
Supplementary Figure 22. siIRF5@EINI treatment improve gait indexes of CIA mice.
Supplementary Figure 23. Gene silencing efficiency of nanoimitators in vivo.
Supplementary Figure 24. Gating strategy used to identify synovial macrophages in rheumatoid arthritis tissue.
Supplementary Figure 25. Secreted level of CXCL1 in synovial tissue isolated from inflamed joints of treated mice.
Supplementary Figure 26. Flow cytometry analysis of M1 and M2 macrophage populations in synovial tissue from different treatment groups.
Supplementary Figure 27. Immunohistochemical staining for CD31 and TRAP.
Supplementary Figure 28. Gating strategies for multicolor flow cytometry analysis.
Supplementary Table 1. List of primers used for real-time PCR.
Supplementary Table 2. Summary of antibodies.

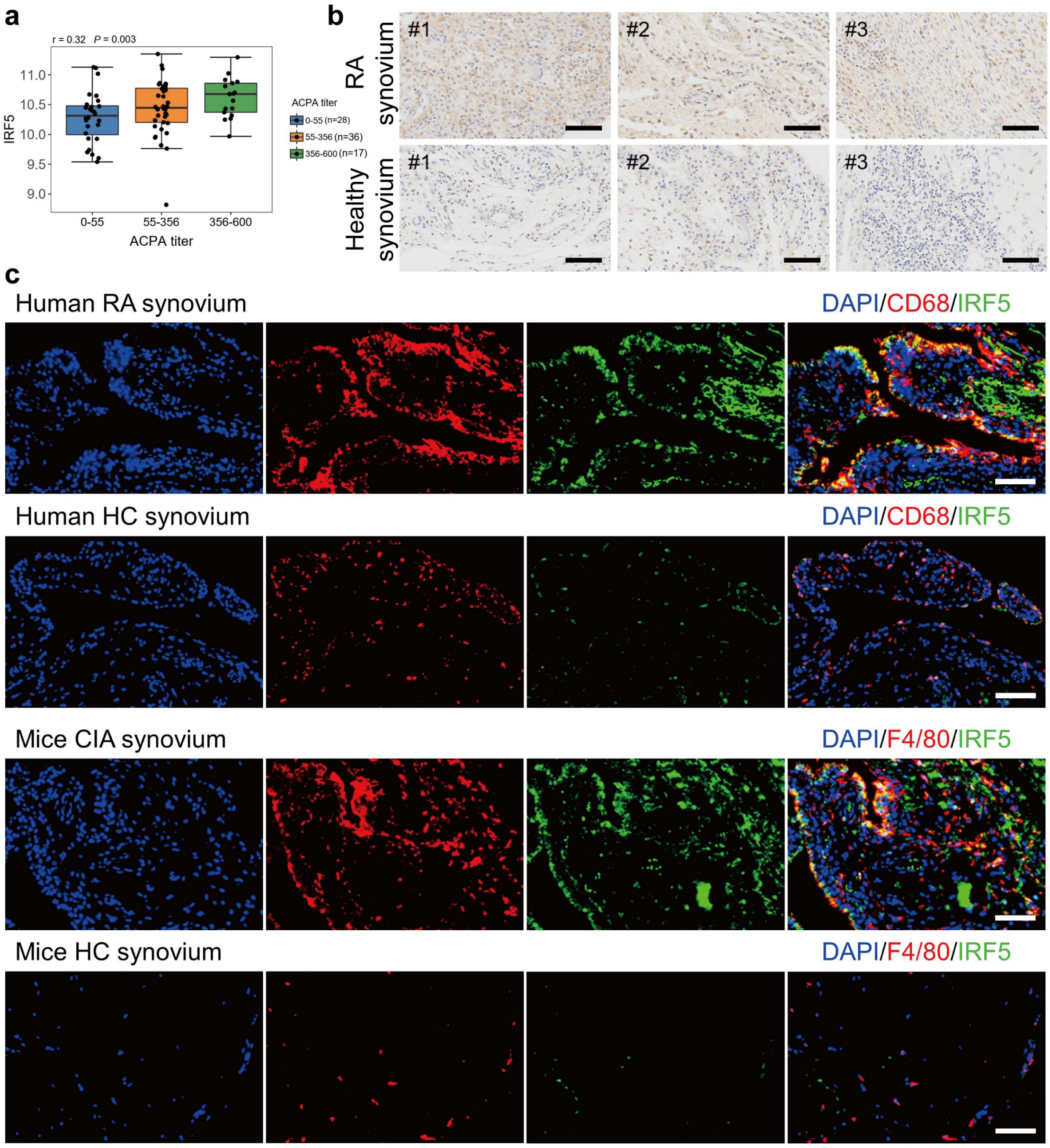

Supplementary Figure 1. IRF5 expression profiles in synovium. a, The positive correlation between the IRF5 gene expression levels and the anti-citrullinated protein antibody (ACPA) titer in the RA synovium (r = 0.32, *P* = 0.003). ACPA titer range 0-55 (*n* = 28 biologically independent samples), ACPA titer range 55-356 (*n* = 36 biologically independent samples), and ACPA titer range 356-600 (*n* = 17 biologically independent samples). The box and whisker plots present a five-number summary: minima, lower quartile, center, upper quartile and maxima. **b**, Immunohistochemical staining of IRF5 in the synovium from RA patients and healthy donors (*n* = 3 independent experiments). Scale bar = 50 μm. **c**, Immunostained sections of IRF5 in synovium from RA patients, CIA mice and healthy control (HC). IRF5 is green and

$CD68^+$ or $F4/80^+$ macrophages are colored red. Nuclei are stained with DAPI (blue) (*n* = 3 independent experiments). Scale bar = 50 µm.

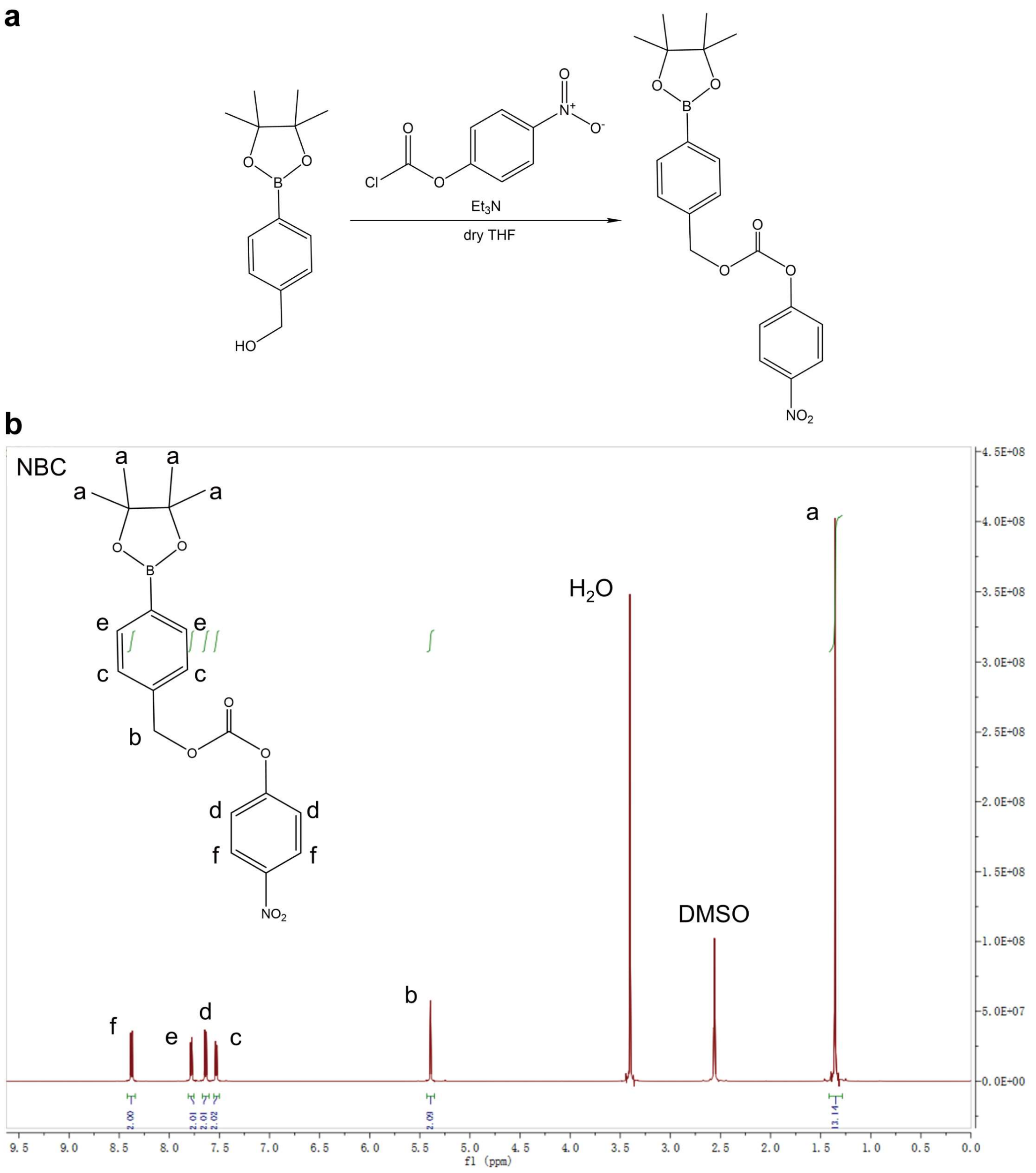

Supplementary Figure 2. Synthesis of NBC. a, Synthetic NBC scheme. **b**, ^{1}H NMR spectrum of NBC in DMSO-d6.

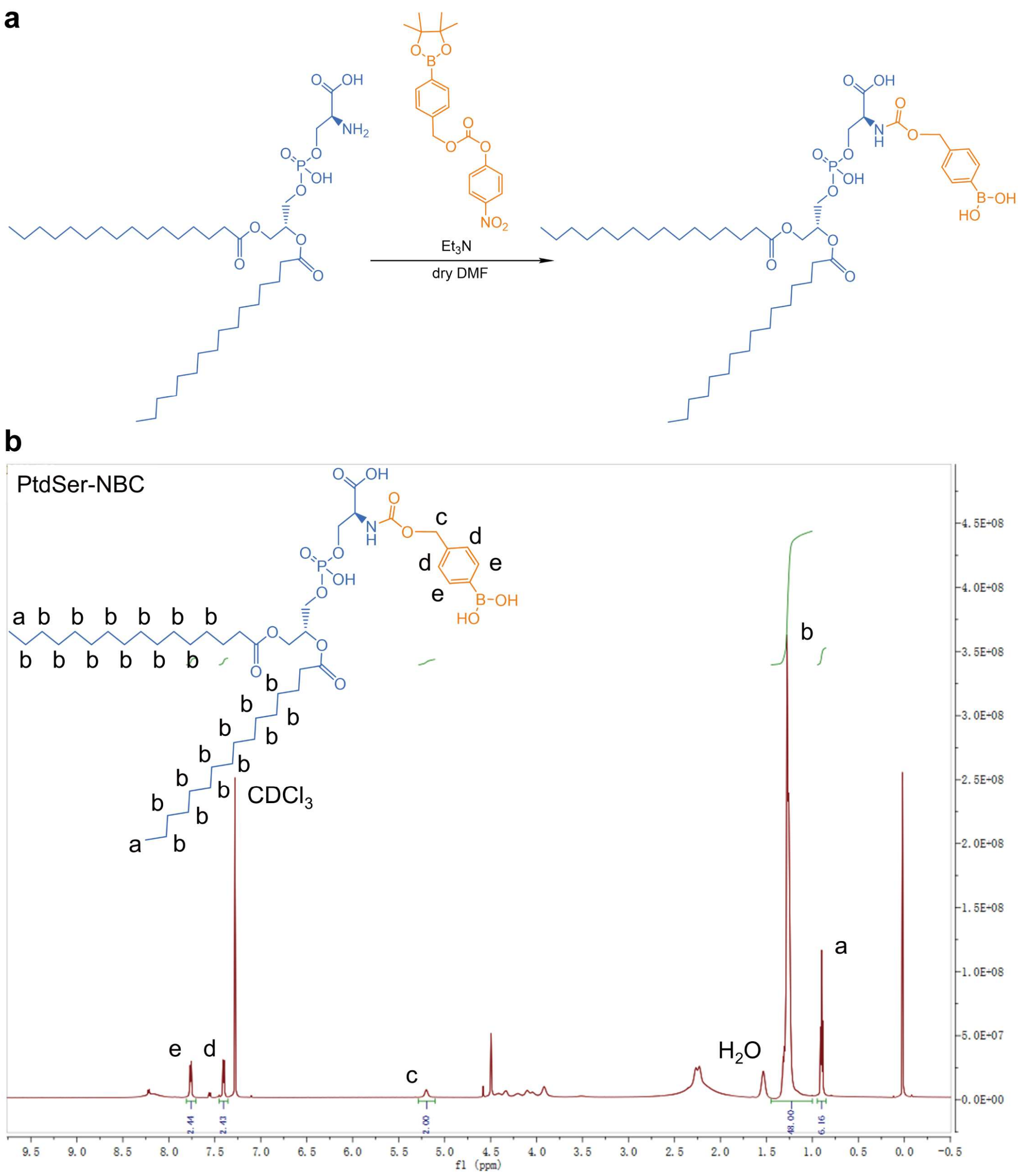

Supplementary Figure 3. Synthesis of PtdSer-NBC. a, Synthetic route developed to produce PtdSer-NBC. **b**, ^{1}H NMR spectrum of PtdSer-NBC in $CDCl_3$.

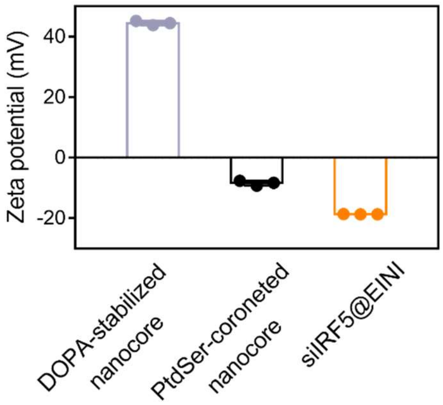

Supplementary Figure 4. Zeta potential of the DOPA-stabilized nanocore, PtdSer-coroneted nanocore and siIRF5@EINI. Data are presented as the mean ± s.d. (n = 3 independent experiments).

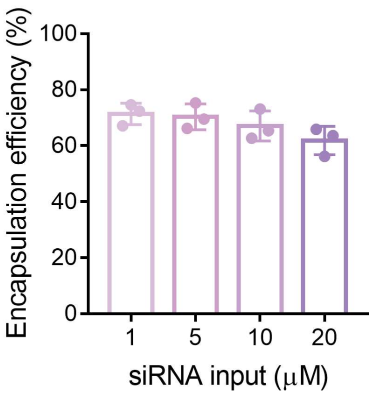

Supplementary Figure 5. Encapsulation efficiency of siRNA inside siIRF5@EINI at various siRNA inputs. Data are presented as the mean ± s.d. (n = 3 independent experiments).

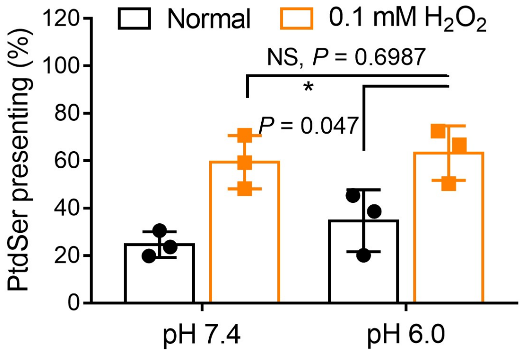

Supplementary Figure 6. Flow cytometry analysis of PtdSer-presenting. The nanoimitator was resuspended in PBS at pH 7.4 or pH 6.0 and incubated with or without H_2O_2 (0.1 mM). Data are presented as the mean ± s.d. (n = 3 independent experiments). (exact P values: P = 0.6987, P = 0.047); *P < 0.05. NS, not significant. Statistical significance was determined by a two-sided Student’s t test. Source data are provided as a Source Data file.

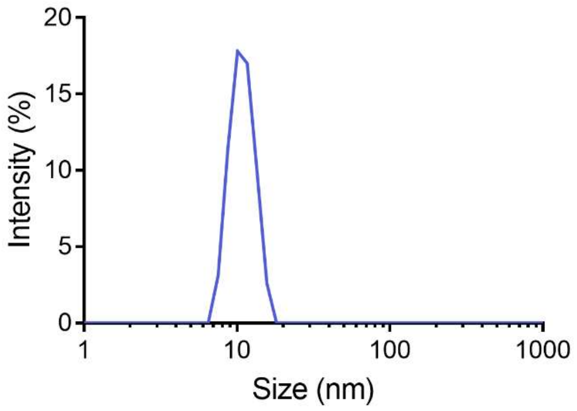

Supplementary Figure 7. DLS data on the nanoimitator after induction of degradation under acidic conditions (pH 5.0).

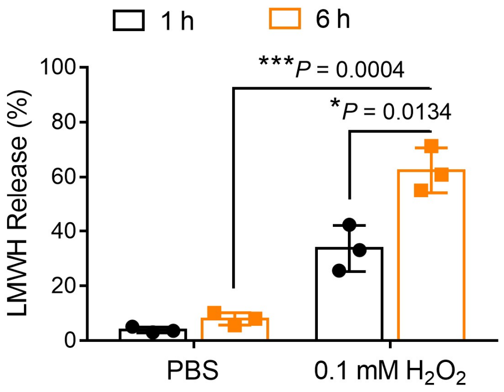

Supplementary Figure 8. The release of LMWH from the nanoimitator in PBS with or without 0.1 mM H_2O_2. Data are presented as the mean ± s.d. (n = 3 independent experiments). (exact P values: P = 0.0004, P = 0.0134); *P < 0.05, ***P < 0.001. Statistical significance was determined by a two-sided Student's t test. Source data are provided as a Source Data file.

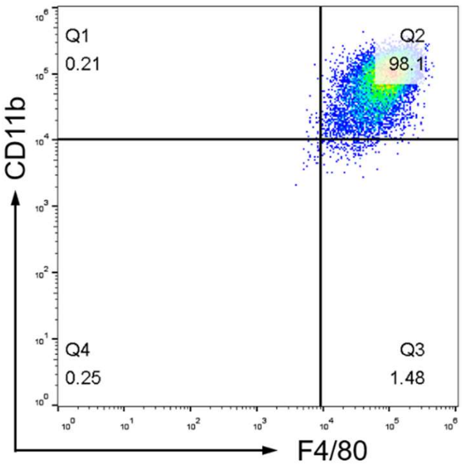

Supplementary Figure 9. The expression levels of CD11b and F4/80 in BMDMs were analyzed by flow cytometry.

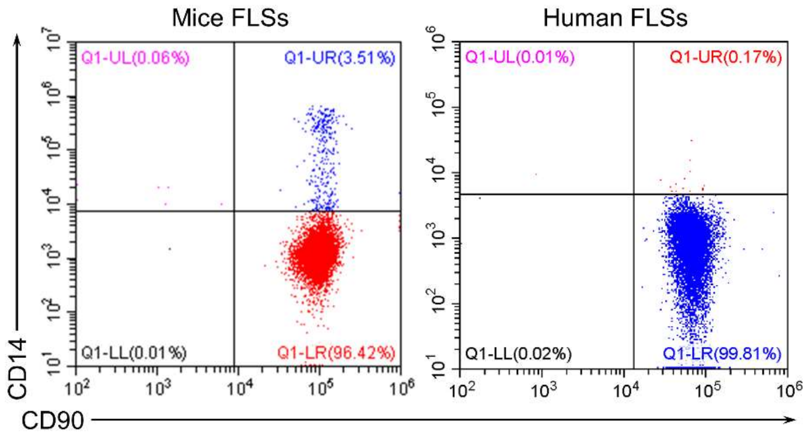

Supplementary Figure 10. Characterization FLSs. Flow cytometry using antibodies against the fibroblast marker CD90 and the macrophage marker CD14 led to the identification of pure FLSs. Mouse FLSs (>90% $CD90^+$, <4% $CD14^+$) and human FLSs (>90% $CD90^+$, <1% $CD14^+$) were positive for the fibroblast marker CD90 and negative for the macrophage marker CD14.

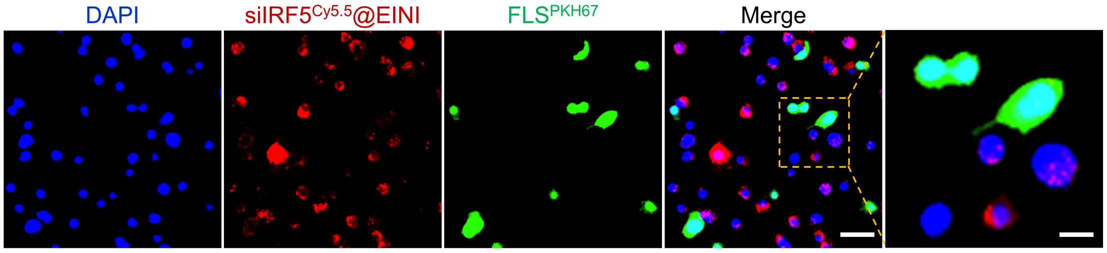

Supplementary Figure 11. Cell-specific uptake of the nanoimitator by human peripheral blood macrophages in a coculture pattern with human FLSs (n = 3 independent experiments). Scale bar, 50 µm and 10 µm.

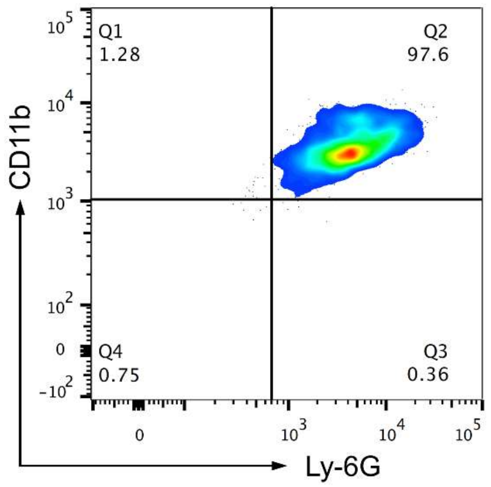

Supplementary Figure 12. Flow cytometric analysis of the purity of neutrophils doubly stained with FITC-conjugated Ly-6G and PerCP-Cy5.5-conjugated CD11b antibodies.

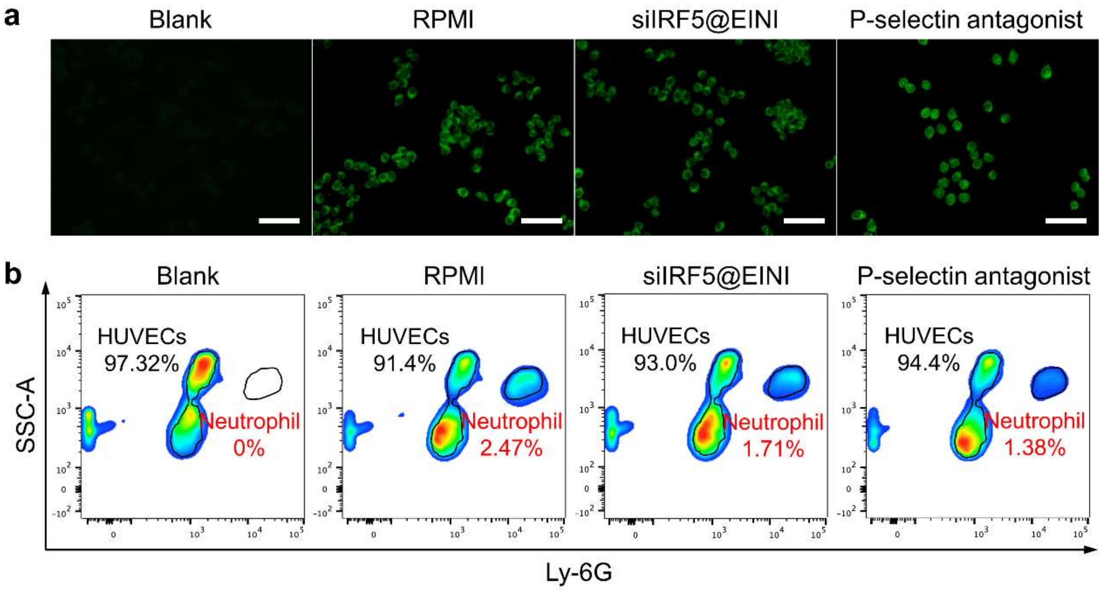

Supplementary Figure 13. Effective intervention by nanoimitator on inflammatory neutrophils. a, Evaluation of neutrophil adhesion to a HUVEC monolayer after incubation with the nanoimitator. Scale bars, 50 μm. **b**, FACS analysis of neutrophil adhesion to HUVECs. All values are expressed as the mean ± s.d. (*n* = 3 independent experiments).

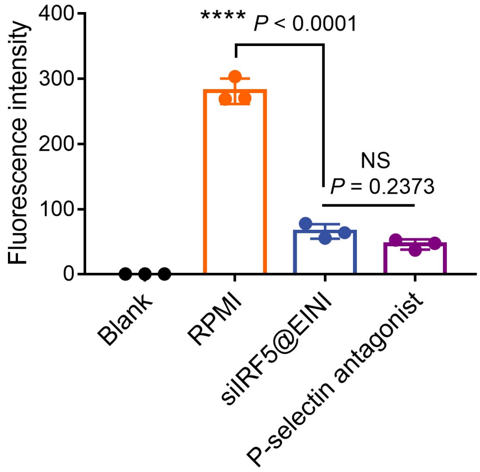

Supplementary Figure 14. Quantitative analysis of neutrophils adhered to HUVEC monolayers. Neutrophils were stained with calcein-AM. The cells in each well were lysed with 2 mL dimethyl sulfoxide (DMSO), and their fluorescence intensities were detected by a fluorospectro-photometer at Ex = 495 nm and Em = 515 nm. Data are reported as mean ± s.d. (n = 3 independent experiments). (exact P values: P = 6.60191E-08, P = 0.2373); ****$P < 0.0001$. NS, not significant. Statistical analysis was performed using one-way ANOVA with Tukey's post hoc test. Source data are provided as a Source Data file.

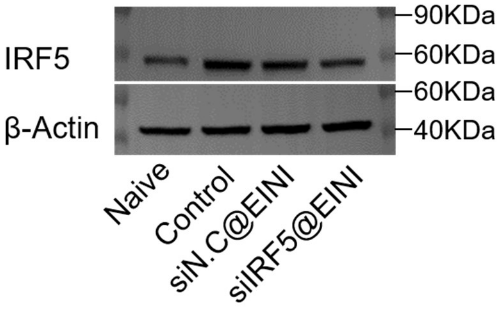

Supplementary Figure 15. IRF5 expression in macrophages treated with different formulations, as detected by Western blotting (n = 3 independent experiments).

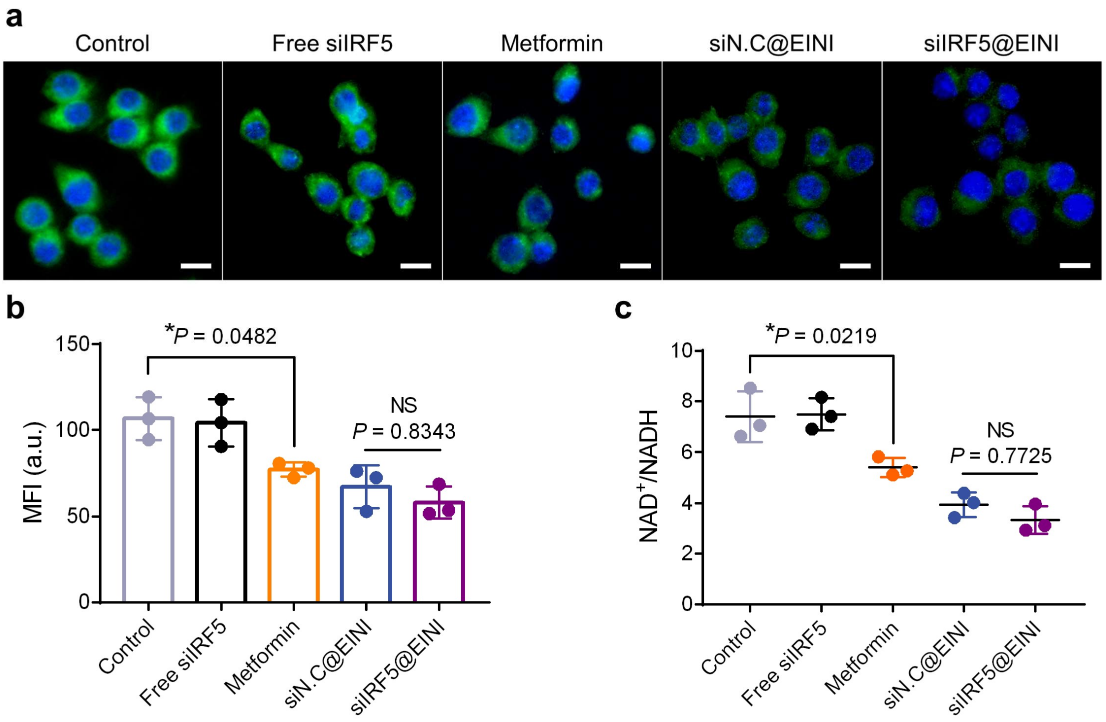

Supplementary Figure 16. Metformin reprograms mitochondrial metabolism in macrophages and acts as mitochondrial complex I (CI) inhibitor that directly inhibits CI-derived ROS. a, Fluorescence images and quantification result (**b**) of intracellular ROS generation in macrophages stained with DCFH-DA. The scale bars represent 20 µm. All values are expressed as the mean ± s.d. (n = 3 biologically independent animals). (exact P values: $P = 0.0482$, $P = 0.8343$); *$P < 0.05$. NS, not significant. **c**, The levels of oxidized and reduced nicotinamide adenine dinucleotide (NAD^+/NADH) were measured in macrophages using a colorimetric assay. All values are expressed as the mean ± s.d. (n = 3 biologically independent animals). (exact P values: $P = 0.0219$, $P = 0.7725$); *$P < 0.05$. NS, not significant. Statistical analysis was performed using one-way ANOVA with Tukey's post hoc test for **b**, **c**. Source data are provided as a Source Data file.

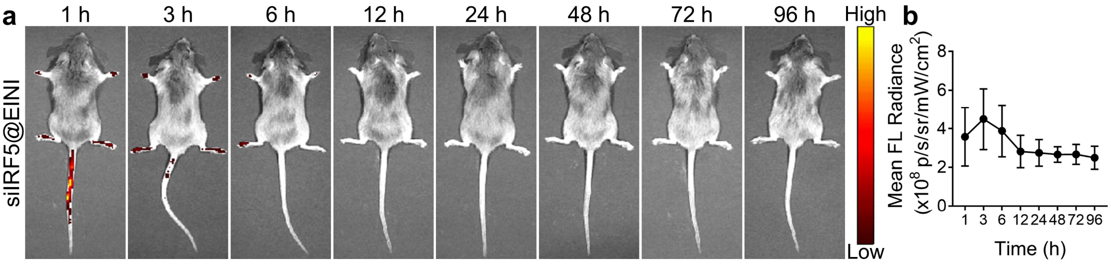

Supplementary Figure 17. In vivo biodistribution of nanoimitators in non-arthritic DBA/1J mice. **a**, Fluorescence images of non-arthritic DBA/1J mice receiving an intravenous injection of Dir-labeled nanoimitator. **b**, Fluorescence intensity of the nanoimitator in the joints of the non-arthritic DBA/1J mice as a function of time. Data are the mean ± s.d. (n = 3 independent experiments).

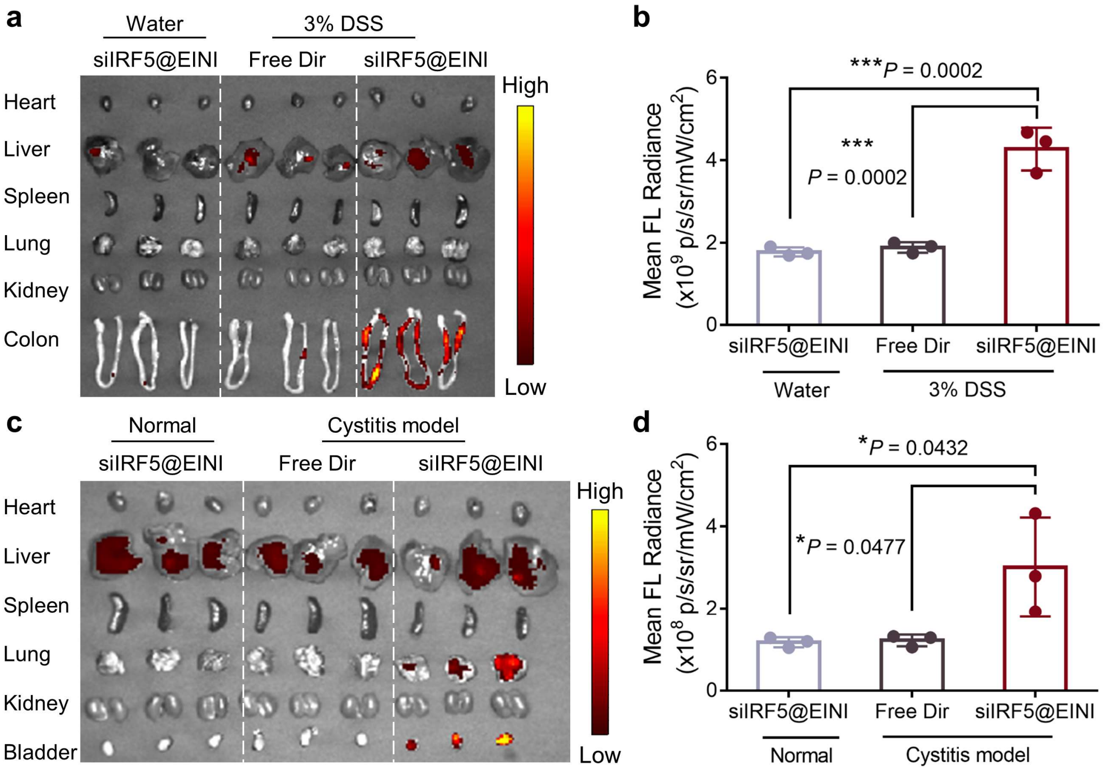

Supplementary Figure 18. Inflammation-targeted drug delivery of the nanoimitator. **a**, **b** At 6 h after treating animals with nanoimitator, their organs were imaged by in vivo imaging system (IVIS) (**a**) and quantitative analysis of the fluorescence intensity in the colon were performed (**b**). Data are presented as the mean ± s.d. (n = 3 biologically independent animals per group). (exact P values: $P = 0.0002$, $P = 0.0002$); ***$P < 0.001$. **c**, **d** In vivo fluorescence imaging shows the accumulation of the DiR-labeled nanoimitator in the bladder (**c**) and quantitative analysis of the fluorescence intensity (**d**). Data are presented as the mean ± s.d. (n = 3 biologically independent animals per group). (exact P values: $P = 0.0432$, $P = 0.0477$); *$P < 0.05$. Statistical analysis was performed using one-way ANOVA with Tukey's post hoc test for **b**, **d**. Source data are provided as a Source Data file.

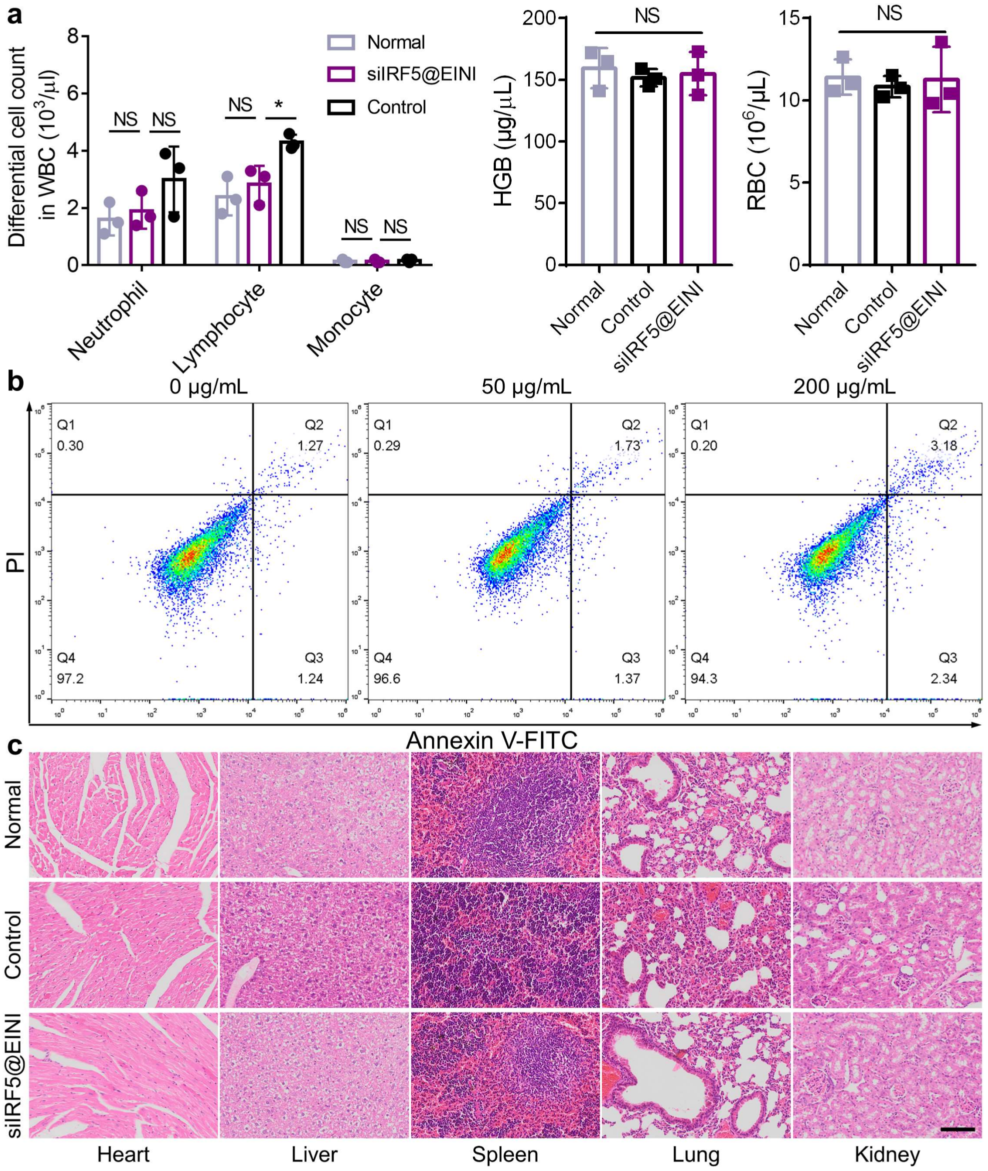

Supplementary Figure 19. Systemic toxicity evaluation of siIRF5@EINI. a, During the experimental cycle, mice were followed up to the time of sacrifice on day 60. White blood cells (WBC) counts, including counts of neutrophils, lymphocytes and monocytes were assessed, and the changes in hemoglobin (HGB) and red blood cells (RBC) were measured. Data are presented as the mean ± s.d. (n = 3 independent experiments). (exact P values: neutrophil: $P = 0.8977$, $P = 0.302$; lymphocyte: $P = 0.6249$, $P = 0.0398$; monocyte: $P > 0.9999$, $P = 0.7684$; HGB: $P = 0.9286$; RBC: $P = 0.9913$); $*P < 0.05$, NS, not significant. **b**, Apoptosis of T cells extracted from the spleens of mice that received i.v. injections of siIRF5@EINI during the experimental cycle. Mice injected with PBS were used as a negative control. **c**, Histological sections of major organs on the 60th day. Scale bar, 50 µm. (n = 3 biologically independent

animals). Statistical analysis was performed using one-way ANOVA with Tukey's post hoc test for **a**. Source data are provided as a Source Data file.

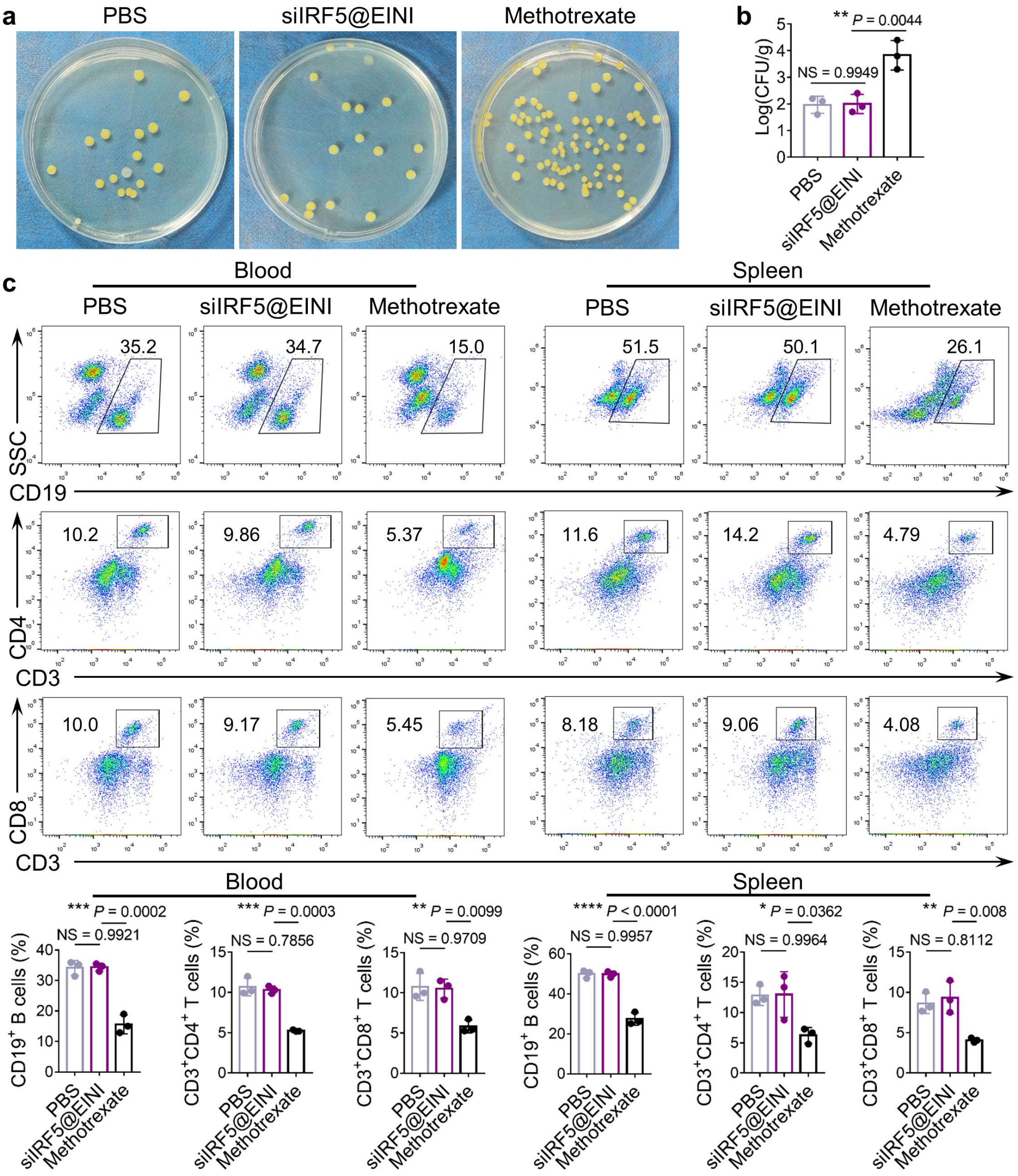

Supplementary Figure 20. Assessment of the impact of the nanoimitator on the immune responses of mice to *Candida albicans* infection. a, Ex vivo culture of C. albicans with spleen lysates from the mice subjected to different treatments. **b**, The quantitative results of each groups splenic bacteria load. Data are presented as the mean ± s.d. (*n* = 3 independent experiments). (exact *P* values: *P* = 0.9949, *P* = 0.0044); ***P* < 0.01, NS, not significant. **c**, $CD19^+$, $CD4^+$and $CD8^+$ cell percentage in blood and spleen of DBA/1J mice treated with the nanoimitator, PBS or methotrexate. Data are presented as the mean ± s.d. (*n* = 3 independent experiments). (exact *P* values: CD19: *P* = 0.9921, *P* = 0.0002; CD4: *P* = 0.7856, *P* = 0.0003; CD8: *P* = 0.9709, *P* = 0.0099; CD19: *P* = 0.9957, *P* = 5.46175E-05; CD4: *P* = 0.9964, *P* = 0.0362; CD8: *P* = 0.8112,

$P = 0.008$); *$P < 0.05$, **$P < 0.01$, ***$P < 0.001$, ****$P < 0.0001$. NS, not significant. Statistical analysis was performed using one-way ANOVA with Tukey's post hoc test for **b**, **c**. Source data are provided as a Source Data file.

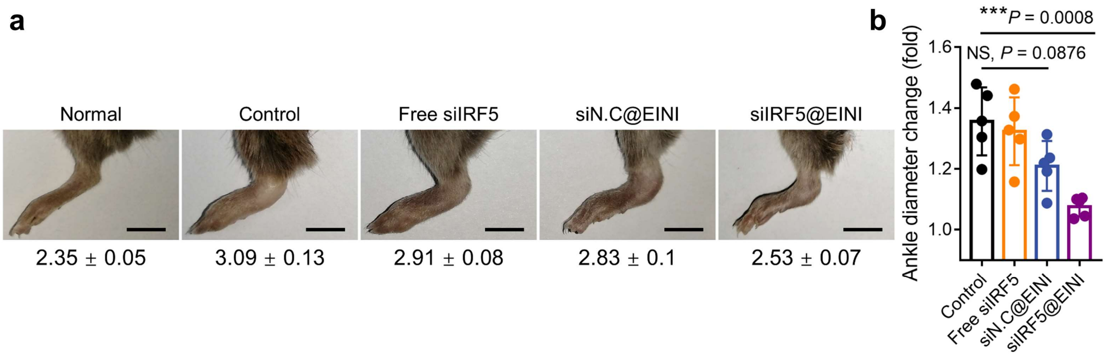

Supplementary Figure 21. Ankle diameter change. **a**, Representative lesion images from macroscopic observation of hind paws from different treatment groups. Scale bar, 3 mm. (n = 5 biologically independent animals). **b**, Change in the average hind ankle in diameter on day 60 after CIA induction compared to that on day 0. Data are presented as the mean ± s.d. (n = 5 biologically independent animals per group). (exact P values: P = 0.0876, P = 0.0008); ***P < 0.001. NS, not significant. Statistical analysis was performed using one-way ANOVA with Tukey's post hoc test for **b**. Source data are provided as a Source Data file.

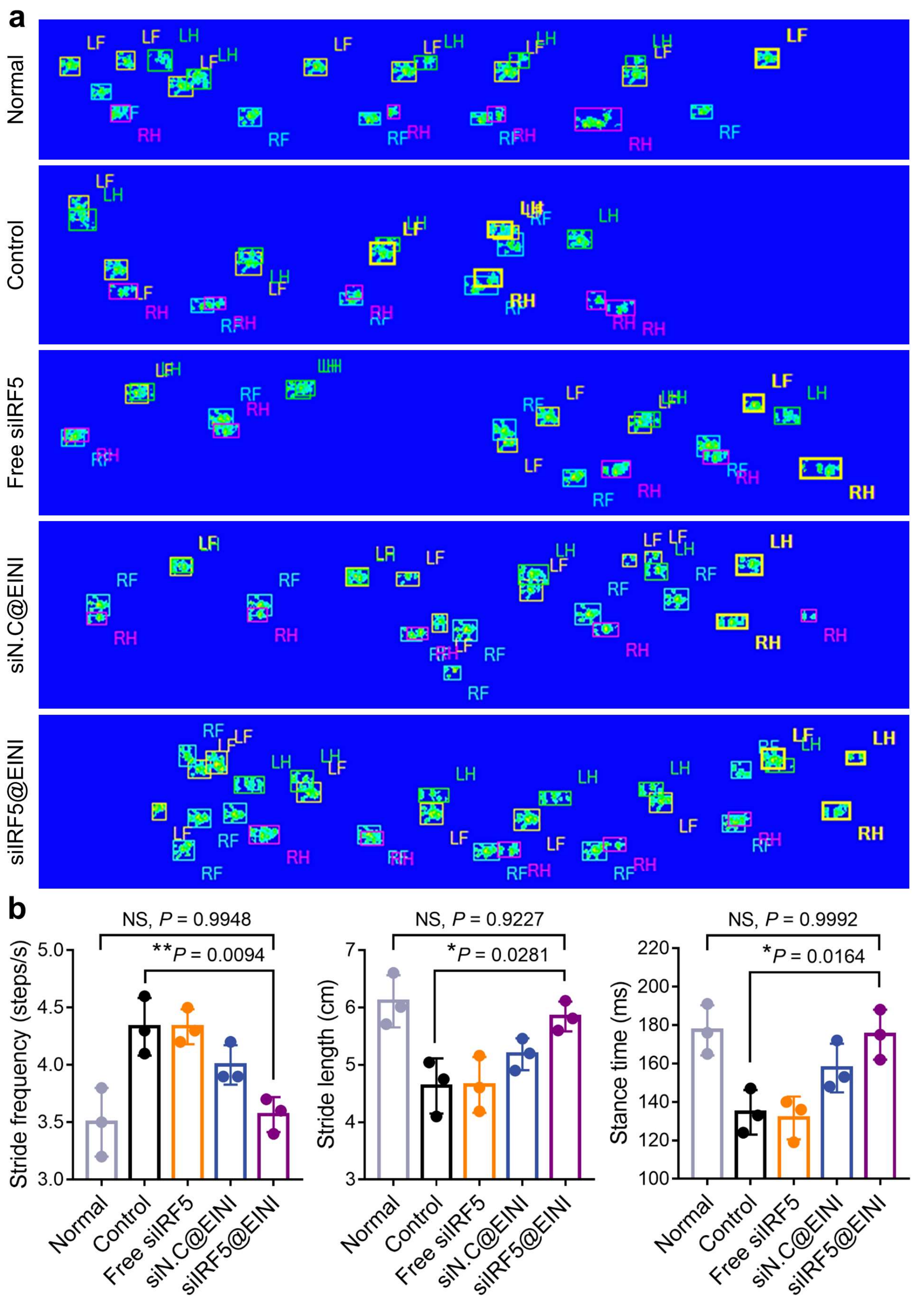

Supplementary Figure 22. siIRF5@EINI treatment improved the gait indexes of CIA mice. a, Representative images of signals from the footprint assay. **b**, Main indexes used for gait detection in mice. Stride frequency (the average number of times a paw contacts the belt per second) increased linearly with increasing clinical score. Stride length (the distance between initial contact of the same paw in a complete stride)

progressively decreased with increasing clinical scores. The stance time (the weight-bearing portion of the stride in which the paw remains in contact with the belt) decreased progressively with increasing clinical scores. Data are presented as the mean ± s.d. (n = 3 independent experiments). (exact P values: Stride frequency: $P = 0.9948$, $P = 0.0094$; Stride length: $P = 0.9227$, $P = 0.0281$; Stance time: $P = 0.9992$, $P = 0.0164$); *$P < 0.05$, **$P < 0.01$. NS, not significant. Statistical analysis was performed using one-way ANOVA with Tukey's post hoc test for **b**. Source data are provided as a Source Data file.

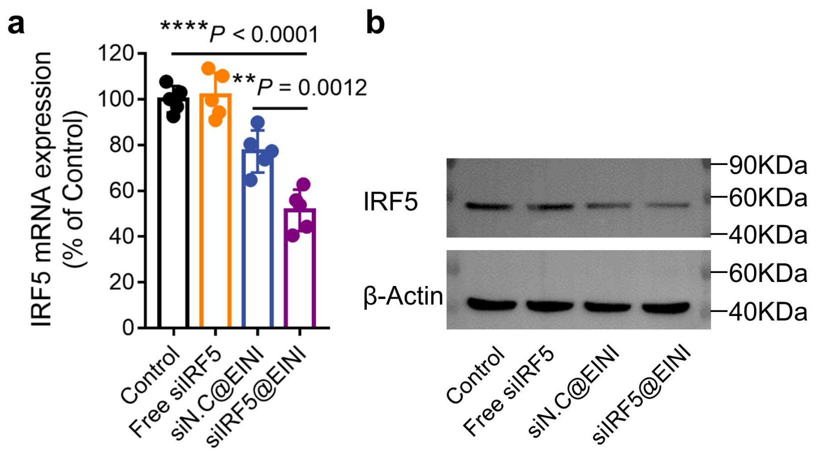

Supplementary Figure 23. Gene silencing efficiency of nanoimitators in vivo. a, Quantification of IRF5 expression in synovial macrophages after each treatment. Data are presented as the mean ± s.d. (*n* = 5 biologically independent animals). (exact *P* values: *P* = 7.33448E-07, *P* = 0.0012); ***P* < 0.01, *****P* < 0.0001. **b**, Western blot images of IRF5 protein levels in the synovial macrophages from groups treated with different formulations (*n* = 3 independent experiments). Statistical analysis was performed using one-way ANOVA with Tukey's post hoc test for **a**. Source data are provided as a Source Data file.

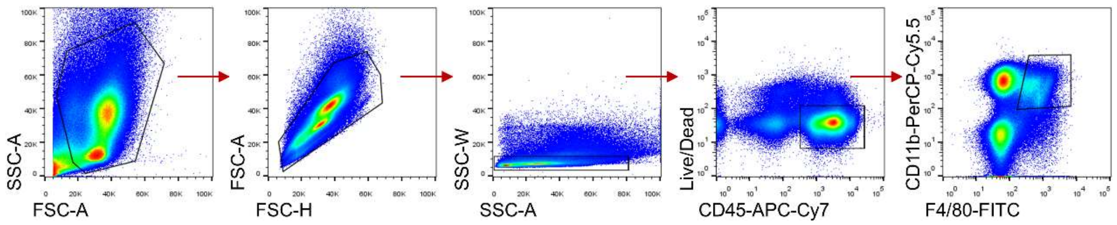

Supplementary Figure 24. Gating strategy used to identify synovial macrophages in rheumatoid arthritis tissue.

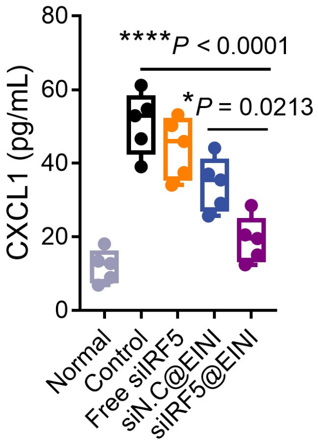

Supplementary Figure 25. Secreted level of CXCL1 in synovial tissue isolated from inflamed joints of treated mice. Data are presented as the mean ± s.d. (n = 5 biologically independent animals). (exact P values: P = 5.862E-06, P = 0.0213); *P < 0.05, ****P < 0.0001. Statistical analysis was performed using one-way ANOVA with Tukey's post hoc test. In the box plots, the top and bottom edges of boxes indicate the first and third quartiles, respectively; the center lines indicate the medians; and the ends of whiskers indicate the maximum and minimum values, respectively. Source data are provided as a Source Data file.

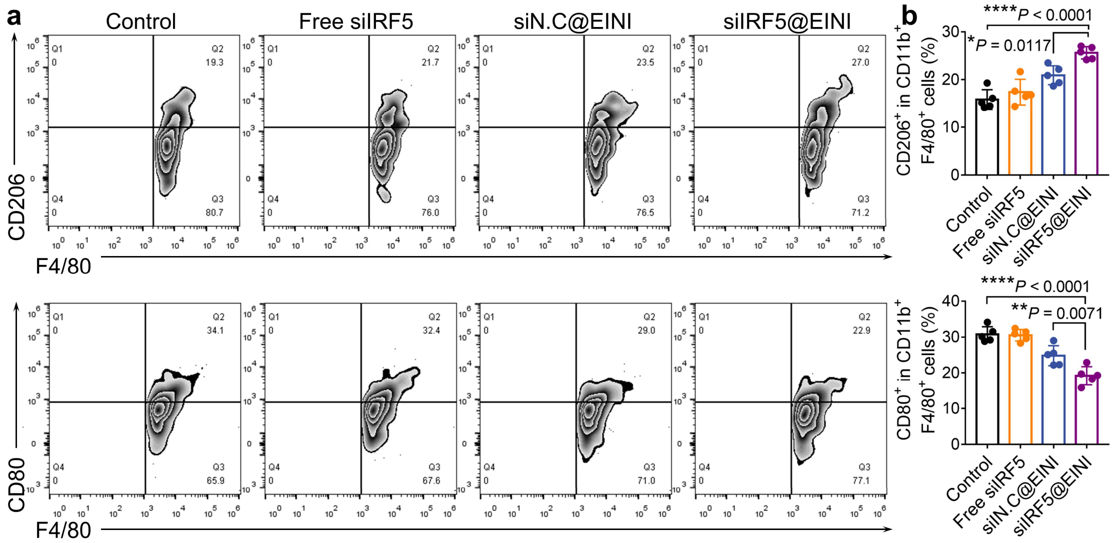

Supplementary Figure 26. Flow cytometry analysis of M1 and M2 macrophage populations in synovial tissue from different treatment groups. Representative flow cytometric analysis images (**a**) and relative quantification of M2-like macrophages ($CD206^+$) and M1-like macrophages ($CD80^+$) gating on $CD11b^+F4/80^+CD45^+$ cells (**b**). For the gating strategy for macrophages analysis refer to Supplementary Fig. 28. Data are presented as the mean ± s.d. (n = 5 biologically independent samples). (exact P values: CD206: P = 6.95112E-06, P = 0.0117; CD80: P = 3.77978E-06, P = 0.0071); *$P < 0.05$, **$P < 0.01$, ****$P < 0.0001$. Statistical analysis was performed using one-way ANOVA with Tukey's post hoc test for **b**. Source data are provided as a Source Data file.

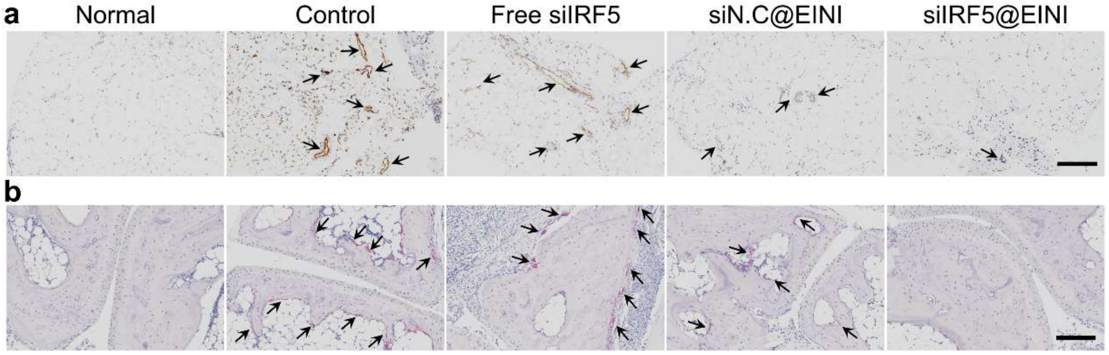

Supplementary Figure 27. Immunohistochemical staining for CD31 and TRAP. a, Representative immunohistochemical staining images of the angiogenesis marker CD31 in the synovium of mice receiving the indicated treatment. The arrows indicated typical $CD31^+$ microvessels. Scale bar = 100 μm. (*n* = 5 biologically independent animals per group). **b**, Immunohistochemical analyses of TRAP-stained osteoclasts in the joint tissues of mice receiving the indicated treatment. The arrows indicated typical TRAP-stained osteoclasts. Scale bar = 100 μm. (*n* = 5 biologically independent animals).

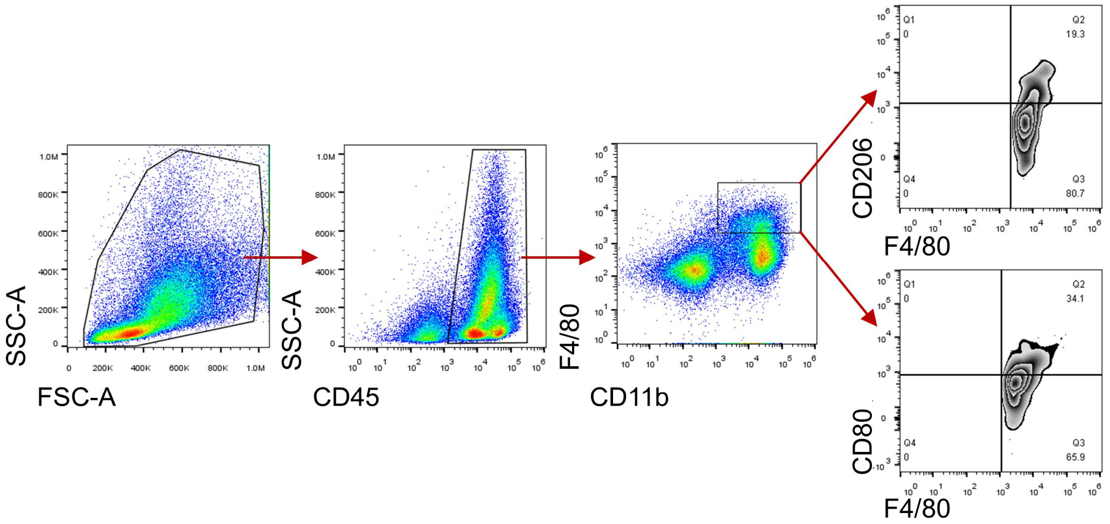

Supplementary Figure 28. Gating strategies for multicolor flow cytometry analysis. The flow cytometric gating strategy of synovial macrophages corresponding to Supplementary Fig. 26a.

Supplementary Table 1. List of primers used for real-time PCR

IRF5	Forward	5’-CATTCAGCGGGAAGTCAAGA-3’
	Reverse	5’-TGTCTGCCGACCAAGAAAGC-3’
CXCL1	Forward	5’-CACCCAAACCGAAGTCATAGC-3’
	Reverse	5’-GGGGACACCTTTTAGCATCTTT-3’
iNOS	Forward	5'-AGCCAAGCCCTCACCTACTT-3’
	Reverse	5'-CTCTGCCTATCCGTCTCGTC-3’
TNF	Forward	5’-TGGAACTGGCAGAAGAGGCAC-3’
	Reverse	5’-AGGGTCTGGGCCATAGAACTGA-3’
Arg1	Forward	5’-TGCTTAGCTCTGTCTGCTTTGC-3’
	Reverse	5’-GAACACGGCAGTGGCTTTAAC-3’
CD206	Forward	5’-GGAGGCTGATTACGAGCAGT-3’
	Reverse	5’-CATAGGAAACGGGAGAACCA-3’
β-actin	Forward	5'-CTACAATGAGCTGCGTGTGG-3’
	Reverse	5'-CAGGTCCAGACGCAGGATGGC-3’

Supplementary Table 2. Summary of antibodies

Antibody	Application	Catalog No.; Supplier	Dilution
Rabbit anti-CD68	IF	76437T; Cell Signaling	1:400
Rabbit anti-F4/80	IF	Ab6640; Abcam	1:200
Rabbit anti-iNOS	IF	Ab178945; Abcam	1:250
Rabbit anti-CD206	IF	Ab300621; Abcam	1:50
Rabbit anti-IRF5	Western	10547-1-AP; proteintech	1:5000
Rabbit anti-IRF5	IHC	76983S; Cell Signaling	1:1000
Rabbit anti- Myeloperoxidase	IHC	ab188211; Abcam	1:8000
FITC anti-mouse F4/80	FC	123107; BioLegend	1:200
PerCP-Cy5.5 anti-mouse CD11b	FC	101227; BioLegend	1:100
FITC anti-mouse Ly-6G	FC	127605; BioLegend	1:100
APC/Cyanine7 anti-mouse CD45	FC	103115; BioLegend	1:100
PE/Dazzle 594 anti-mouse CD80	FC	104738; BioLegend	1:200
PE anti-mouse CD206	FC	141705; BioLegend	1:100
PE anti-mouse CD45	FC	103106; BioLegend	1:200
APC/Cyanine7 anti-mouse CD3	FC	100222; BioLegend	1:200
APC anti-mouse CD4	FC	100412; BioLegend	1:200
PE/Cyanine7 anti-mouse CD8a	FC	100722; BioLegend	1:200
APC anti-mouse CD19	FC	152409; BioLegend	1:200
PE anti-mouse CD90	FC	Ab24904; Abcam	1:100
FITC anti-mouse CD14	FC	123307; BioLegend	1:100
PE anti-human CD90	FC	328109; BioLegend	1:100
FITC anti-human CD14	FC	325603; BioLegend	1:100

*IF: Immunofluorescence; IHC: Immunohistochemistry; FC: Flow Cytometry;
