# Supplementary material for: Remodeling articular immune homeostasis with an efferocytosis-informed nanoimitator mitigates rheumatoid arthritis in mice
Source: Nat Commun. 2023 Feb 13;14:817. doi: 10.1038/s41467-023-36468-2 (PMC9925448; doi:10.1038/s41467-023-36468-2)
Supplement: Supplementary file 3 — Reporting Summary [file 41467_2023_36468_MOESM3_ESM.pdf]

nature portfolio

Corresponding author(s): Yuankai Zhang, Xinyi Jiang

Last updated by author(s): Dec 16, 2022

Reporting Summary

Nature Portfolio wishes to improve the reproducibility of the work that we publish. This form provides structure for consistency and transparency in reporting. For further information on Nature Portfolio policies, see our Editorial Policies and the Editorial Policy Checklist.

Statistics

For all statistical analyses, confirm that the following items are present in the figure legend, table legend, main text, or Methods section.

n/a	Confirmed	
☐	☒	The exact sample size (*n*) for each experimental group/condition, given as a discrete number and unit of measurement
☐	☒	A statement on whether measurements were taken from distinct samples or whether the same sample was measured repeatedly
☐	☒	The statistical test(s) used AND whether they are one- or two-sided<br>*Only common tests should be described solely by name; describe more complex techniques in the Methods section.*
☐	☒	A description of all covariates tested
☐	☒	A description of any assumptions or corrections, such as tests of normality and adjustment for multiple comparisons
☐	☒	A full description of the statistical parameters including central tendency (e.g. means) or other basic estimates (e.g. regression coefficient) AND variation (e.g. standard deviation) or associated estimates of uncertainty (e.g. confidence intervals)
☐	☒	For null hypothesis testing, the test statistic (e.g. *F*, *t*, *r*) with confidence intervals, effect sizes, degrees of freedom and *P* value noted<br>*Give P values as exact values whenever suitable.*
☒	☐	For Bayesian analysis, information on the choice of priors and Markov chain Monte Carlo settings
☒	☐	For hierarchical and complex designs, identification of the appropriate level for tests and full reporting of outcomes
☒	☐	Estimates of effect sizes (e.g. Cohen's *d*, Pearson's *r*), indicating how they were calculated

Our web collection on statistics for biologists contains articles on many of the points above.

Software and code

Policy information about availability of computer code

Data collection	GE Amersham Imager 600 RGB, Applied Biosystems7900 Real-Time PCR system, BD Accuri C6 Plus, Beckman Coulter Gallios, Beckman Coulter CytoFLEX S, Beckman Coulter Moflo Astrios EQ, Carl Zeiss LSM780, Olympus IX73 + DP80, PerkinElmer IVIS Spectrum, PerkinElmer Quantum GX2, Olympus VERSUS120, ChemDraw Ultra 7.0.
Data analysis	FlowJo (X 10.0.7r2) was used for flow cytometry analysis, ImageJ (1.52v) was used to merge the fluorescent image, R4.1.0. was used for bioinformatics analysis. The quantification of µCT was performed using CTAn software (version 1.13.2.1, SkyScan). GraphPad Prism 7 and Microsoft Excel 2016 were used for data analysis and plotting.

For manuscripts utilizing custom algorithms or software that are central to the research but not yet described in published literature, software must be made available to editors and reviewers. We strongly encourage code deposition in a community repository (e.g. GitHub). See the Nature Portfolio guidelines for submitting code & software for further information.

Data

Policy information about availability of data

All manuscripts must include a data availability statement. This statement should provide the following information, where applicable:

- Accession codes, unique identifiers, or web links for publicly available datasets
- A description of any restrictions on data availability
- For clinical datasets or third party data, please ensure that the statement adheres to our policy

All data generated from this study are available within the Article, Supplementary Information or Source Data file. IRF5 mRNA expression level in patients with RA

retrieved from the ArrayExpress (https://www.ebi.ac.uk/arrayexpress/) under Accession code E-MTAB-6141. Source data are provided with this paper.

Human research participants

Policy information about studies involving human research participants and Sex and Gender in Research.

Reporting on sex and gender	Sex- and gender-based analyses are not performed in the study. Studies involving human research participants are immunohistochemical staining using human synovial biopsy tissue for evaluation the IRF5 expression in synovium from RA patients relative to healthy donors synovial tissues.
Population characteristics	Human synovial tissue sections were obtained from ACPA+ RA patients (1 male and 2 females; average age: 66.8 years) and healthy donors (2 males and 1 female; average age: 56.3 years) from the Department of Orthopaedic Surgery at Shandong Provincial Hospital Affiliated to Shandong First Medical University.
Recruitment	RA patients were recruited at the Department of Rheumatology and Immunology, healthy individuals were recruited at the Department of Trauma Surgery. All patients signed an informed consent. There was no bias in recruitment and this was not a clinical trial.
Ethics oversight	All samples were collected using a standard protocol approved under the Review Board protocol of Shandong First Medical University (Approve NO.2019-272).

Note that full information on the approval of the study protocol must also be provided in the manuscript.

Field-specific reporting

Please select the one below that is the best fit for your research. If you are not sure, read the appropriate sections before making your selection.

[x] Life sciences [ ] Behavioural & social sciences [ ] Ecological, evolutionary & environmental sciences

For a reference copy of the document with all sections, see nature.com/documents/nr-reporting-summary-flat.pdf

Life sciences study design

All studies must disclose on these points even when the disclosure is negative.

Sample size	Sample size are provided in the figure legends for each experiment and reasonable sample sizes were chosen to ensure they are sufficient for statistical comparison between different groups. For in vitro experiments and analysis, a sample size of 3 was used to detect a significant difference between different groups. For analysing the inflamed joints targeting effects of EINI, 3 mice from each group were housed for in vivo imaging, 3 mice from each group were euthanized for histological staining. The in vivo efficacy studies were performed with 5 mice per group. Details regarding sample size of all experiments are provided in the Methods section and figure legends. Samples sizes for in vivo experiments were chosen empirically based upon preliminary rheumatoid arthritis therapy experiments. Input and approval from Shandong University's Institutional Animal Care and Use Committee were also considered. Sample sizes for in vitro experiments were also chosen empirically based upon preliminary experiments to achieve statistical significance.
Data exclusions	No data was excluded from the analyses.
Replication	Experiments were replicated independently for at least 3 times. The number of replicates is detailed in the caption of each figures in the main manuscript and supplementary information files. Experiments were repeated and experimental findings were reproducible.
Randomization	Samples were randomly allocated to corresponding experimental groups. Organisms were cultured and maintained in the same environment and randomly allocated to each group.
Blinding	Clinical scores and histologic evaluations were performed in a blinded manner. For in vitro cell culture studies blinding was not relevant to this study as all measures were quantifiable by standard cellular or biochemical assays.

Reporting for specific materials, systems and methods

We require information from authors about some types of materials, experimental systems and methods used in many studies. Here, indicate whether each material, system or method listed is relevant to your study. If you are not sure if a list item applies to your research, read the appropriate section before selecting a response.

Materials & experimental systems

n/a	Involved in the study
☐	☒ Antibodies
☐	☒ Eukaryotic cell lines
☒	☐ Palaeontology and archaeology
☐	☒ Animals and other organisms
☒	☐ Clinical data
☒	☐ Dual use research of concern

Methods

n/a	Involved in the study
☒	☐ ChIP-seq
☐	☒ Flow cytometry
☒	☐ MRI-based neuroimaging

Antibodies

Antibodies used

Rabbit antibodies against IRF5 (Cell Signaling Technology; Catalog number: 76983S; 1:1000 dilution for immunohistochemical staining), Rabbit antibodies against IRF5 (proteintech; Catalog number: 10547-1-AP; 1:5000 for western blotting assay), Rabbit monoclonal antibody to Myeloperoxidase (Abcam; Catalog number: ab188211; Clone name: EPR17996; 1:8000 dilution for immunohistochemistry), FITC anti-mouse F4/80 Antibody (BioLegend, Catalog number: 123107; 1:200 dilution for flow cytometry), PerCP-Cy5.5 anti-mouse CD11b Antibody (BioLegend, Catalog number: 101227; 1:100 dilution for flow cytometry), FITC anti-mouse Ly-6G Antibody (BioLegend, Catalog number: 127605; 1:100 dilution for flow cytometry), Rat monoclonal antibody to F4/80 (Abcam; Catalog number: ab6640; Clone name: A3-1; 1:200 dilution for immunofluorescence), Rabbit monoclonal antibody to CD206 (Abcam; Catalog number: ab300621; Clone name: EPR25215-277; 1:50 dilution for immunofluorescence), Rabbit monoclonal antibody to iNOS (Abcam; Catalog number: ab178945; Clone name: EPR16635; 1:250 dilution for immunofluorescence), APC/Cyanine7 anti-mouse CD45 Antibody (BioLegend, Catalog number: 103115; 1:100 dilution for flow cytometry). PE/Dazzle™ 594 anti-mouse CD80 Antibody (BioLegend, Catalog number: 104738; 1:200 dilution for flow cytometry). PE anti-mouse CD206 (MMR) Antibody (BioLegend, Catalog number: 141705; 1:100 dilution for flow cytometry). PE anti-mouse CD45 Antibody (BioLegend, Catalog number: 103106; 1:200 dilution for flow cytometry). APC/Cyanine7 anti-mouse CD3 Antibody (BioLegend, Catalog number: 100222; 1:200 dilution for flow cytometry). APC anti-mouse CD4 Antibody (BioLegend, Catalog number: 100412; 1:300 dilution for flow cytometry). PE/Cyanine7 anti-mouse CD8a Antibody (BioLegend, Catalog number: 100722; 1:200 dilution for flow cytometry). APC anti-mouse CD19 Antibody (BioLegend, Catalog number: 152409; 1:200 dilution for flow cytometry). PE anti-mouse CD90 (Abcam, Catalog number: Ab24904; 1:100 dilution for flow cytometry). FITC anti-mouse CD14 (BioLegend, Catalog number: 123307; 1:100 dilution for flow cytometry). PE anti-human CD90 (BioLegend, Catalog number: 328109; 1:100 dilution for flow cytometry). FITC anti-human CD14 (BioLegend, Catalog number: 325603; 1:100 dilution for flow cytometry). Rabbit anti-CD68 (Cell Signaling Technology; Catalog number: 76437T; 1:400 dilution for immunofluorescence)

Validation

All antibodies were verified by the supplier and validation data are available on the manufacturer's website.
1. Rabbit antibodies against IRF5 have been validated to be used for immunohistochemical staining with western blotting assay and mentioned species reactivity with human. (https://www.cellsignal.cn/products/primary-antibodies/irf-5-e7f9w-rabbit-mab/76983);
2. Rabbit antibodies against IRF5 have been validated to be used for western blotting assay and mentioned species reactivity with mouse. (https://www.ptgcn.com/products/IRF5-Antibody-10547-1-AP.htm);
3. Rabbit monoclonal antibody to Myeloperoxidase has been validated to be used for immunohistochemical staining and mentioned species reactivity with mouse. (https://www.abcam.cn/myeloperoxidase-antibody-epr17996-ab188211.html);
4. FITC anti-mouse F4/80 Antibody has been validated to be used for flow cytometric analysis and mentioned species reactivity with mouse. (https://www.biolegend.com/en-us/search-results/fitc-anti-mouse-f4-80-antibody-4067);
5. PerCP-Cy5.5 anti-mouse CD11b Antibody has been validated to be used for flow cytometric analysis and mentioned species reactivity with mouse. (https://www.biolegend.com/en-us/products/percp-cyanine5-5-anti-mouse-human-cd11b-antibody-4257);
6. FITC anti-mouse Ly-6G Antibody has been validated to be used for flow cytometric analysis and mentioned species reactivity with mouse. (https://www.biolegend.com/en-us/search-results/fitc-anti-mouse-ly-6g-antibody-4775);
7. Rat monoclonal antibody to F4/80 has been validated to be used for immunofluorescence analysis and mentioned species reactivity with mouse. (https://www.abcam.cn/f480-antibody-cia3-1-macrophage-marker-ab6640.html);
8. Rabbit monoclonal antibody to CD206 has been validated to be used for immunofluorescence analysis and mentioned species reactivity with mouse. (https://www.abcam.cn/mannose-receptor-antibody-epr25215-277-ab300621.html);
9. Rabbit monoclonal antibody to iNOS has been validated to be used for immunofluorescence analysis and mentioned species reactivity with mouse. (https://www.abcam.cn/inos-antibody-epr16635-ab178945.html);
10. APC/Cyanine7 anti-mouse CD45 Antibody has been validated to be used for flow cytometric analysis and mentioned species reactivity with mouse. (https://www.biolegend.com/en-us/search-results/apc-cyanine7-anti-mouse-cd45-antibody-2530);
11.PE/Dazzle™ 594 anti-mouse CD80 Antibody has been validated to be used for flow cytometric analysis and mentioned species reactivity with mouse. (https://www.biolegend.com/en-us/products/pe-dazzle-594-anti-mouse-cd80-antibody-10221);
12.PE anti-mouse CD206 (MMR) Antibody has been validated to be used for flow cytometric analysis and mentioned species reactivity with mouse. (https://www.biolegend.com/en-us/products/pe-anti-mouse-cd206-mmr-antibody-7424);
13.PE anti-mouse CD45 Antibody has been validated to be used for flow cytometric analysis and mentioned species reactivity with mouse. (https://www.biolegend.com/en-us/products/pe-anti-mouse-cd45-antibody-100);
14.APC/Cyanine7 anti-mouse CD3 Antibody has been validated to be used for flow cytometric analysis and mentioned species reactivity with mouse. (https://www.biolegend.com/en-us/products/apc-cyanine7-anti-mouse-cd3-antibody-6068);
15.APC anti-mouse CD4 Antibody has been validated to be used for flow cytometric analysis and mentioned species reactivity with mouse. (https://www.biolegend.com/en-us/products/apc-anti-mouse-cd4-antibody-245);
16.PE/Cyanine7 anti-mouse CD8a Antibody has been validated to be used for flow cytometric analysis and mentioned species reactivity with mouse. (https://www.biolegend.com/en-us/products/pe-cyanine7-anti-mouse-cd8a-antibody-1906);
17.APC anti-mouse CD19 Antibody has been validated to be used for flow cytometric analysis and mentioned species reactivity with mouse. (https://www.biolegend.com/en-us/products/apc-anti-mouse-cd19-antibody-13680);

18.PE anti-mouse CD90 has been validated to be used for flow cytometric analysis and mentioned species reactivity with mouse. (https://www.abcam.cn/pe-cd90--thy1-antibody-g7-ab24904.html);
19.FITC anti-mouse CD14 has been validated to be used for flow cytometric analysis and mentioned species reactivity with mouse. (https://www.biolegend.com/en-us/products/fitc-anti-mouse-cd14-antibody-4327);
20.PE anti-human CD90 has been validated to be used for flow cytometric analysis and mentioned species reactivity with human. (https://www.biolegend.com/en-us/products/pe-anti-human-cd90-thy1-antibody-4114);
21.FITC anti-human CD14 has been validated to be used for flow cytometric analysis and mentioned species reactivity with human. (https://www.biolegend.com/en-us/products/fitc-anti-human-cd14-antibody-3951);
22.Rabbit anti-CD68 has been validated to be used for immunofluorescence analysis and mentioned species reactivity with human. (https://www.cellsignal.cn/products/primary-antibodies/cd68-d4b9c-xp-rabbit-mab/76437?site-search type=Products&N=4294956287&Ntt=rabbit+anti-cd68&fromPage=plp);

Eukaryotic cell lines

Policy information about cell lines and Sex and Gender in Research

Cell line source(s)

Bone marrow-derived macrophages (BMDMs) were generated from mouse bone marrow monocytes. Mouse neutrophils (NEs) were collected from whole blood of C57BL/6 mice using a modified Percoll gradient method. Murine fibroblast-like synoviocytes (FLSs) were isolated from knee joint synovium of CIA mice. Human FLSs were isolated from primary synovial tissue obtained from 3 patients with RA who met the American College of Rheumatology (formerly, the American Rheumatism Association) revised criteria and had undergone total joint replacement surgery or synovectomy. Human umbilical vein endothelial cells (HUVECs) were purchased from Institute of Biochemistry and Cell Biology (Shanghai Institutes for Biological Sciences, Chinese Academy of Science).

Authentication

The mature macrophages were stained with PerCP-Cy5.5 conjugated anti-CD11b and FITC conjugated anti-F4/80 antibodies, and then evaluated by flow cytometry. Mouse neutrophils were stained with PerCP-Cy5.5 conjugated anti-CD11b and FITC conjugated Ly-6G antibodies to identify neutrophil cells. Pure FLSs (>90% CD90+/ < 1% CD14+) were identified by flow cytometry using antibodies against the fibroblast marker CD90 and the macrophage marker CD14. The HUVECs cell line was not validated, cell morphology and behavior were consistent with expectations.

Mycoplasma contamination

All cell lines showed negative for mycoplasma contamination.

Commonly misidentified lines
(See ICLAC register)

No commonly misidentified cell lines were used in this study.

Animals and other research organisms

Policy information about studies involving animals; ARRIVE guidelines recommended for reporting animal research, and Sex and Gender in Research

Laboratory animals

DBA/1J mice in collagen-induced arthritis study (male, 6-weeks-old, 18-22g) were used without special care. Bone marrow-derived macrophages were generated from bone-marrow progenitor cells of C57BL/6 mice (male, 7-weeks-old, 22-24g) and used without further modification. Mouse neutrophils were isolated from whole blood of LPS-stimulated C57BL/6 mice (male, 7-weeks-old, 22-24g) and used without further modification. Mice were housed under conditions of a light/dark cycle of 12 h, an ambient temperature of 25 ± 2 °C, and a humidity of 60 ± 10%. Mice were randomized before the experiment.

Wild animals

No wild animals were involved.

Reporting on sex

Male

Field-collected samples

The study did not involve samples collected from field.

Ethics oversight

All animal procedures were performed in accordance with the Guidelines for Care and Use of Laboratory Animals of Shandong University and experiments were approved by the Animal Ethics Committee of the School of Shandong University.

Note that full information on the approval of the study protocol must also be provided in the manuscript.

Flow Cytometry

Plots

Confirm that:

- [x] The axis labels state the marker and fluorochrome used (e.g. CD4-FITC).
- [x] The axis scales are clearly visible. Include numbers along axes only for bottom left plot of group (a 'group' is an analysis of identical markers).
- [x] All plots are contour plots with outliers or pseudocolor plots.
- [x] A numerical value for number of cells or percentage (with statistics) is provided.

Methodology

Sample preparation	i: For the in vitro cellular uptake analysis, BMDMs were cultured with each formulation, and cellular uptake of nanoimitator was assessed quantitatively via the fluorescent property of Cy5.5 labeled siIRF5.<br>ii: The BMDMs were collected after detachment with 0.05% trypsin-EDTA and centrifugation at 400g for 5 min, and then the BMDMs were stained with PerCP-Cy5.5 conjugated anti-CD11b and FITC conjugated anti-F4/80 antibodies. The induction of mature macrophages was evaluated by flow cytometry.<br>iii: Mouse neutrophils were isolated from whole blood of LPS stimulated C57BL/6 mice by Percoll density gradient centrifugation. The neutrophils were stained with PerCP-Cy5.5 conjugated anti-CD11b and FITC conjugated Ly-6G antibodies to identify neutrophil cells.<br>iv: The blood and spleens of mice were collected. Red blood cells were lysed with ACK lysing buffer. The remaining cells in the samples were pelleted by a centrifugation at 300g for 5 min, and then the antibody staining was performed according to the manufacturer's instructions.<br>v: Fresh synovial tissues were digested with 1 mg/ml type I collagenase in HBSS, and incubated at 37 °C for 5% CO_2 in a humidified atmosphere for 30-45 min. Disaggregated tissue elements were passed through a 70 µm cell strainer. Then, the antibody staining was performed according to the manufacturer's instructions.
Instrument	BD Accuri C6 Plus, Beckman Coulter Gallios, Beckman Coulter CytoFLEX S and Beckman Coulter Moflo Astrios EQ were used for flow cytometry data collection.
Software	FlowJo (X 10.0.7r2) was used for data analysis.
Cell population abundance	Synovial macrophages were FACS-sorted on Beckman Coulter Moflo Astrios EQ and the purity was at least 92%.
Gating strategy	i: The mature bone marrow-derived macrophages were gated via the inclusion of singlets, CD11b+, and F4/80+ cells.<br>ii: The mouse neutrophils were gated via the inclusion of singlets, CD11b+, and Ly-6G+ cells.<br>iii: Phenotypic changes in the synovial macrophage subpopulation were gated via the inclusion of singlets, CD45+, CD11b+, F4/80+, CD206+ or CD80+. For dead cell exclusion, Fixable Viability Dye was used (Sigma-Aldrich).<br>iv: Synovial macrophages from the synovium were defined as singlets, CD45+, CD11b+, F4/80+. For dead cell exclusion, Fixable Viability Dye was used (Sigma-Aldrich).

☒ Tick this box to confirm that a figure exemplifying the gating strategy is provided in the Supplementary Information.
